# Biocompatibility Investigation of Hybrid Organometallic Polymers for Sub-Micron 3D Printing via Laser Two-Photon Polymerisation

**DOI:** 10.3390/ma12233932

**Published:** 2019-11-27

**Authors:** Evaldas Balčiūnas, Nadežda Dreižė, Monika Grubliauskaitė, Silvija Urnikytė, Egidijus Šimoliūnas, Virginija Bukelskienė, Mindaugas Valius, Sara J. Baldock, John G. Hardy, Daiva Baltriukienė

**Affiliations:** 1 Institute of Biochemistry, Life Sciences Centre, Vilnius University, 10257 Vilnius, Lithuania; ev.balciunas@gmail.com (E.B.); nadezda.dreize@gmail.com (N.D.); monika.grub@gmail.com (M.G.); urniksi@gmail.com (S.U.); egidijus.simoliunas@gmail.com (E.Š.); virginija.bukelskiene@bchi.vu.lt (V.B.); mindaugas.valius@bchi.vu.lt (M.V.); 2 Department of Chemistry, Lancaster University, Lancaster LA1 4YB, UK; s.baldock@lancaster.ac.uk; 3 Materials Science Institute, Lancaster University, Lancaster LA1 4YB, UK

**Keywords:** bioactive surfaces, biomaterials, hybrid organometallic polymers, laser two-photon polymerisation, tissue engineering

## Abstract

Hybrid organometallic polymers are a class of functional materials which can be used to produce structures with sub-micron features via laser two-photon polymerisation. Previous studies demonstrated the relative biocompatibility of Al and Zr containing hybrid organometallic polymers in vitro. However, a deeper understanding of their effects on intracellular processes is needed if a tissue engineering strategy based on these materials is to be envisioned. Herein, primary rat myogenic cells were cultured on spin-coated Al and Zr containing polymer surfaces to investigate how each material affects the viability, adhesion strength, adhesion-associated protein expression, rate of cellular metabolism and collagen secretion. We found that the investigated surfaces supported cellular growth to full confluency. A subsequent MTT assay showed that glass and Zr surfaces led to higher rates of metabolism than did the Al surfaces. A viability assay revealed that all surfaces supported comparable levels of cell viability. Cellular adhesion strength assessment showed an insignificantly stronger relative adhesion after 4 h of culture than after 24 h. The largest amount of collagen was secreted by cells grown on the Al-containing surface. In conclusion, the materials were found to be biocompatible in vitro and have potential for bioengineering applications.

## 1. Introduction

The concept of growing human replacement parts in the lab has been around for several decades [1,2]. Researchers have used different approaches for the engineering of artificial tissues—from allogeneic or xenogeneic tissue decellularisation [3] to approaches based on additive manufacturing [4], the latter of which offers a route to the generation of an optimal scaffold for a specific tissue type and patient which has significant potential for economic/health/societal impacts.

Laser two-photon polymerisation (LTPP) is a 3D fabrication technique capable of producing materials with fine details in their structures at sub-micron resolutions [5,6]. Various materials can be used to make 3D structures using this technique including derivatives of natural polymers (e.g., hyaluronic acid [7], gelatin [8]) and synthetic ones (e.g., derivative of polyethylene glycol [9] and SU-8 [10]).

The LTPP technique is highly versatile and allows several materials to be used in the same sample [11,12]. Compared to other fabrication techniques like stereo-lithography [13], fused deposition modelling [14,15] and selective laser sintering [16], LTPP is the only technique that allows for resolutions below the diffraction limit of light to be fabricated [17]. In addition, LTPP allows for fine-tuning of structural motifs as opposed to randomised porous structures obtained by other means, like template-casting [18] or particulate-leaching [19]. A group of interesting materials for LTPP are hybrid organometallic polymers [20]. To date, materials based on Al [21], Ge [22], Ti [23], V [24] and Zr [25] have been shown to be structurable using LTPP systems. An in vitro biocompatibility screening study showed that Al and Ti hybrids supported a comparable number of cells to glass, while the Zr-based hybrid exceeded the biocompatibility of all the other surfaces [21]. An in vivo study of our Zr-based hybrids showed them to be relatively biocompatible when implanted in rabbit muscle and that they did not cause inflammation or foreign body reaction as demonstrated by hematoxylin and eosin staining [26].

Cell-extracellular matrix interactions are among the most important processes to attenuate in attempting to recreate structurally and functionally viable tissue constructs analogous to natural tissues (where the resident cells adhere to the ECM and take part in remodelling it over time). For many applications, it is important to have materials that support a native-like cellular response and integration. To understand the influence of the metals contained in the organometallic hybrids on cellular behaviour, we have investigated the process of adhesion and adhesion-associated kinase expression, as well as collagen secretion of primary rat myogenic cells grown on these surfaces. We believe that this work will prove useful for tissue engineering researchers focusing on artificial scaffold-based tissue remodelling techniques in that the materials investigated in this work are highly biocompatible, precisely structurable at sub-micron resolutions and simple to prepare.

## 2. Materials and Methods

### 2.1. Material Synthesis

The hybrid organometallic polymers were synthesised according to published protocols [21,25] Briefly, the Al-based material was prepared by dissolution of aluminium isopropoxide (AIP, ≥98%, Merck, Kenilworth, NJ, USA) in toluene (ACS, ISO, Reag. Ph Eur, Merck, Kenilworth, NJ, USA). In parallel, 3-(trimethoxysilyl)propyl methacrylate (MAPTMS, 98%, Sigma-Aldrich, St. Louis, MO, USA) was hydrolysed using HCl (0.1 M, Applichem, Darmstadt, Germany). Methacrylic acid (MAA, 99%, Sigma-Aldrich, USA) was then added to the solution of aluminium isopropoxide in toluene at a 1:1 molar ratio and subsequently, hydrolysed MAPTMS was added to the mixture at a 1:1:4 AIP:MAA:MAPTMS molar ratio. Finally, 1% of photoinitiator (4,4′-bis(diethylamino)benzophenone, Sigma-Aldrich, St. Louis, MO, USA) was added to the weight of AIP, MAA and 3-(trihydroxysilyl)propyl methacrylate (the product of MAPTMS hydrolysis) and stirred, while shielding from ambient light to prevent undesired crosslinking.

The Zr-based material was prepared in an analogous manner, with molar ratios of zirconium (IV) propoxide, MAA and MAPTHS being 1:1:4 with 1% of photoinitiator by weight (excluding solvents).

### 2.2. 2D Sample Preparation

To secure polymer bonding to glass, MAPTMS-treated glass slides were prepared according to a protocol adopted from Kapyla et al. [27]. Briefly, circular 12 mm diameter borosilicate glass slides (Thermo Fisher Scientific, Waltham, MA, USA) were washed in ethanol, then immersed in a mixture of MAPTMS/ethanol/acetic acid/water overnight and finally washed in ethanol in an ultrasonic bath (EMAG, Mörfelden-Walldorf, Germany).

The hybrid materials were then spin-coated @ 3000 rpm for 30 s per sample and left at room temperature in the dark overnight for the solvents to evaporate. The samples were then polymerised using a UV lamp (UV-C, G15W T8, Sylvania, London, UK) for at least 5 h at around 20 cm distance, corresponding to about 1.8 mW/cm^2^. The samples were sterilised under UV for at least one hour on each side, subsequently washed in sterile PBS to remove residual initiator and low molecular weight components. They were then immersed in sterile growth medium prior to cell culture.

### 2.3. 3D Sample Fabrication via Laser Two-Photon Polymerisation

For laser two-photon polymerisation, a droplet of a hybrid material was placed on a glass slide and then left in a fume hood to evaporate overnight while covered from ambient light. After 24 h such samples were used for structure fabrication on a commercially available Nanoscribe^®^ Photonic Professional GT system (Nanoscribe GmbH, Eggenstein-Leopoldshafen, Germany) that is based at the Department of Chemistry at Lancaster University. A 63X 1.4 NA oil immersion lens was used to focus the laser beam into the polymer. Stereolithography (STL) flies were downloaded from the internet [28] and sliced using proprietary Nanoscribe software (DeScribe 2.3.3). Hatching and slicing distances in X, Y and Z coordinates were set to 100 nm. The laser scanning speed was set to 10 mm/s and the laser power was in the range of 20% to 100% (corresponding to 10–50 mW). After polymerization, the Al based hybrid material was developed in toluene for at least 15 min, followed by air-drying. The structures were then sputter-coated at a thickness of 10 nm using a Q150 RS coater (Quorum Technologies, Lewes, UK) and subsequently observed using a SEM (JSM 7800F, JEOL, Tokyo, Japan) operating at 10–15 kV at the Department of Chemistry at Lancaster University.

### 2.4. Collagen Adsorption and Secretion Assays

A protocol for assessing the concentration of collagen was adapted from Tullberg-Reinert and Jundt [29] with slight modifications. The protocol used is based on in situ staining of cell monolayers using Sirius Red. This dye has strong interactions with collagen types I and III, but weak interaction with collagen type IV.

First, a solution of collagen type I (1 mg/mL in 0.1 N acetic acid, Sigma-Aldrich, St. Louis, MO, USA) was added to the sample and incubated for 1 h at 37 °C. Then, the collagen solution was gently replaced with fixating Bouin’s solution (15 mL saturated aqueous picric acid (Sigma-Aldrich, St. Louis, MO, USA) with 5 mL 35% formaldehyde (Sigma-Aldrich, St. Louis, MO, USA) and 1 mL glacial acetic acid (Sigma-Aldrich, St. Louis, MO, USA), incubated for 1 h and then washed using PBS. The samples were then transferred to new wells with a solution of Sirius Red (1 mg/mL in picric acid) for 1 h under mild rocking conditions (30 rpm). Afterwards, the samples were washed with 0.01 N hydrochloric acid to remove unbound dye. The dye was then dissolved in 0.2–0.3 mL of 0.1 N sodium hydroxide by shaking at room temperature for 30 min. Non-coated samples were stained and measured in the same way and their signals were subtracted from collagen-coated sample signals prior to analysis.

The optical density of the solution was measured using a Varioskan Flash microplate reader (Thermo Fisher Scientific, Waltham, MA, USA) at 550 nm. For reference, a calibration curve was prepared by using known concentrations of collagen type I in order to link them to the absorbance of Sirius Red at 550 nm (Figure S1 in Supplementary Materials). A total of 3 independent experiments were carried out and the results are presented as averages +/- standard errors.

Collagen synthesis was assessed in an analogous way to adsorption. The cells were seeded on the test surfaces at a density of 20,000 cells/mL/sample in 24 well tissue culture plates. After 1, 7 and 14 days of incubation, the samples were fixated using Bouin’s solution, then dyed using a solution of Sirius Red (Sigma-Aldrich, St. Louis, MO, USA) and subsequently washed with a solution of HCl (Sigma-Aldrich, St. Louis, MO, USA). Finally, the dye was dissolved in a solution of NaOH (Merck, Kenilworth, NJ, USA) and measured spectrophotometrically against pure NaOH, and the amount of collagen was determined from the calibration curve. 3 independent experiments were performed with 3 repetitions per material within each experiment.

### 2.5. Cell Isolation, Culture and Characterisation

A Wistar rat was euthanised and a small piece of skeletal muscle tissue was removed. The experiments were approved by License of Animal Research Ethics Committee (Lithuania) No. G2-39, 03/08/2016. Large blood vessels were separated, then skeletal muscle tissue was minced and incubated in a solution of EDTA-trypsin (Gibco, Thermo Fisher Scientific, Waltham, MA, USA) with collagenase and hyaluronidase (0.5% and 0.3%, respectively, Sigma-Aldrich, St. Louis, MO, USA) for 30 min at 37 °C under mild shaking conditions. The resulting cell suspension was centrifuged, mixed with growth medium and seeded to tissue culture plates. The growth medium was replaced every 3–4 days and after 5 passages the cells were cloned by serial dilution. A highly proliferative colony forming unit was selected as a cell source, multiplied in vitro and banked in liquid nitrogen for further use.

The cells were cultured in Iscove’s modified Dulbecco’s medium (IMDM), supplemented with 10% Foetal bovine serum (Gibco, Thermo Fisher Scientific, Waltham, MA, USA) and Penicillin-Streptomycin (100 U/mL and 100 μg/mL, Gibco, Thermo Fisher Scientific, Waltham, MA, USA). They were subcultured every 3–4 days, by detaching with EDTA-trypsin and resuspending in fresh medium. The cells were grown in an incubator (Thermo Fisher Scientific, Waltham, MA, USA) at 37 °C with 5% CO_2_.

For characterization, cells were grown in 30 mm diameter Petri dishes with glass slides on the bottom, fixed with 4% paraformaldehyde for 15 min. Then the cells were washed with PBS and incubated with 0.2% Triton X-100 in PBS for 15 min to permeabilise the membranes. After blocking in 1% BSA in PBS, the cells were incubated with primary antibodies against CD34, C45 (both from Abcam, Cambridge, UK), Myf5 and c-kit (both from Thermo Fisher Scientific, Waltham, MA, USA) overnight according to manufacturer’s instructions at 4 °C. The samples were then rinsed with 1% BSA in PBS and incubated with Cy3-conjugated secondary antibodies (Merck, Kenilworth, NJ, USA). Analysis was performed using an Olympus IX71 (Olympus, Tokyo, Japan) fluorescence microscope.

### 2.6. Analysis of Cell Viability

AO/EB staining distinguishes cells into four categories: live (bright green, intact nucleus), early apoptotic (bright green, but fragmented nucleus), late apoptotic (orange, fragmented nucleus) and necrotic (bright orange, intact nucleus). Cells were cultured on the hybrid polymer and glass surfaces for 24, 48, 72 and 96 h. Then, the growth medium with any unattached cells was collected and the monolayer of cells were treated with EDTA-trypsin and then collected as well. All of them were subsequently stained by acridine orange and ethidium bromide (Sigma-Aldrich, St. Louis, MO, USA) as described in [30]. The cells were analysed using a BD FACSCanto™ II (BD Biosciences, San Jose, CA, USA) flow cytometer, registering 10,000 events per sample and time point. Green fluorescence of acridine orange was detected using FITC channel and red fluoresce of ethidium bromide was detected using PE channel. A total of 3 independent experiments were performed with 3 repetitions per material within each experiment. The cells were split into four categories based on their fluorescence profile.

### 2.7. MTT Assay

MTT assay is based on cellular mitochondrial reductase activity. The absorption measured using this technique is directly proportional to the amount of active enzyme, which gives an idea on the number of viable cells and their metabolic activity.

The cells were seeded at a density of 20,000 cells/mL/sample in 24 well tissue culture plates on the hybrid material and glass surfaces and cultured for 24, 48, 72 and 96 h at 37 °C. The samples were then gently transferred to new tissue culture plates containing a solution of MTT (0.2 mg/mL in PBS) and incubated for 2 h at 37 °C. The solution was subsequently removed and the formazan crystals dissolved in ethanol (96%, Vilniaus degtinė, Vilnius, Lithuania). The absorption was measured using a Varioskan Flash microplate reader (Thermo Fisher Scientific, Waltham, MA, USA) at 570 nm with ethanol as a reference. A total of 3 independent experiments were performed with 3 repetitions per material within each experiment.

### 2.8. Adhesion Strength

The cells were seeded at a density of 100 000 cells/mL/sample in 24 well tissue culture plates and cultured for either 4 or 24 h at 37 °C with 5% CO_2_. At those time points, samples were transferred to new tissue culture plates, and half of the plates were subjected to shaking at 500 rpm for 5 min using a tissue culture plate shaker (Thermomixer Comfort, Eppendorf, Hamburg, Germany). The reference samples (unshaken) were incubated at 37 °C. The number of cells remaining adhered to the surface was measured by replacing the growth medium with 0.1% crystal violet solution in 20% ethanol for 30 min. Then cells were washed with tap water. Before the measurement of absorption, dye was solubilised with 0,1% acetic acid solution in 50% ethanol. Optical density proportional to cell number was measured using a Varioskan Flash microplate reader (Thermo Fisher Scientific, Waltham, MA, USA) at 570 nm and comparing it to analogously dyed, unshaken cell monolayers. A total of 3 independent experiments were performed with 3 repeats per material within each experiment.

### 2.9. Signalling Protein Expression and Phosphorylation

The cells were seeded on the samples at a density of 100,000 cells/mL/sample and cultured for either 4 or 24 h. At these timepoints, the samples were transferred to new tissue culture plates and gently washed with PBS. The PBS was then replaced with a lysis buffer consisting of 8 M urea, 2 M thiourea and 50 mM DTT. Cells were collected from 5 samples for each timepoint by pipetting 5–10 times. The lysates were then centrifuged for 10 min at 20,000 × G at RT. The supernatants of each vial were then transferred to new vials and frozen at −20 °C until further use. A total of 3 independent experiments was carried out.

Protein concentrations were normalised by running an SDS-PAGE gel, staining with Coomassie brilliant blue, taking images using a transilluminator (UVP, Upland, CA, USA) using analysing by ImageJ software. After diluting the highest concentration samples using lysis buffer, the concentration-equalised samples were subjected to gel electrophoresis again at 200 V for 45 min using a BioRad (Hercules, CA, USA) electrophoresis apparatus. The proteins were then transferred to a PVDF membrane (Carl Roth, Karlsruhe, Germany) using a Biometra Fastblot transfer device (Biometra GmbH, Göttingen, Germany) at 25 V and 300 mA. The membranes were blocked using 1% BSA (Sigma-Aldrich, St. Louis, MO, USA) in TBS with 0.1% Tween 20 (Sigma-Aldrich, St. Louis, MO, USA).

The membranes were subsequently treated with primary antibodies against p-Akt (Ser473 and Thr308, Cell Signalling technology, Danvers, MA, USA), Akt (Molecular Probes, Eugene, OR, USA), FAK (BD Biosciences, San Jose, CA, USA) and fluorescently-labelled anti-α-tubulin (Sigma-Aldrich, St. Louis, MO, USA) overnight according to manufacturer’s instructions at 4 °C.

Next day, the membranes were washed three times using wash buffer and incubated with secondary antibodies. FAK and Akt were treated with HRP-conjugated goat anti-mouse (Invitrogen, Carlsbad, CA, USA) and HRP-conjugated anti-rabbit (Invitrogen, Carlsbad, CA, USA) according to manufacturer’s instructions for 1 h. Finally, the membranes were washed again three times using wash buffer. FAK and Akt were detected using by treating with ECL reagent, which upon catalysis by HRP yields chemiluminescence that was detected using a transilluminator (UVP, Upland, CA, USA).

P-Akt (Ser473) and p-Akt (Thr308) were treated with secondary goat-anti-rabbit antibodies conjugated to a fluorescent infrared dye (IRDye 800CW, LI-COR, Lincoln, NE, USA) and detected using an infrared imaging system (Odyssey, LI-COR, Lincoln, NE, USA).

Membrane image analysis was performed using ImageJ software (National Institutes of Health, USA).

### 2.10. Statistical Analysis

All experiments were repeated at least 3 times independently with 3 repetitions within each experiment. The results are presented as averages +/− standard deviations (*n* = 0–5) or errors (*n* = 6+). Statistical significance was assessed using one-way or two-way ANOVA and Tukey’s HSD post-hoc tests using RStudio (RStudio Inc., Boston, MA, USA) and plotted using the ggplot2 package. Statistical significance was considered to be achieved with *p* < 0.05.

## 3. Results and Discussion

### 3.1. Laser Two-Photon Polymerisation

**Figure 1:**
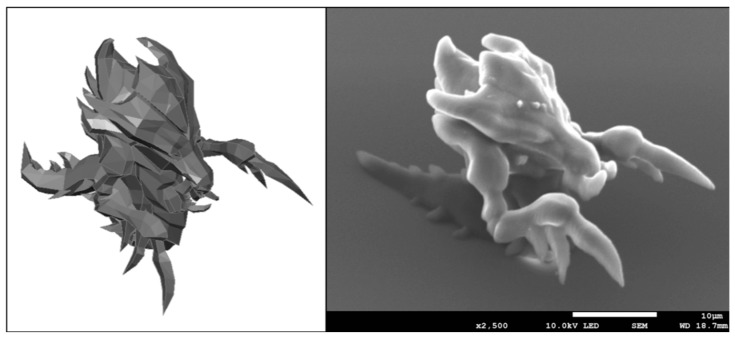
Right, A 3D micro-structure reminiscent of Zerg fabricated using a commercially available Nanoscribe Photonic Professional GT system. The scale bar corresponds to 10 microns. Left, A CAD model of the structure.

As mentioned in the introduction and methods, the possibility of structuring the Al-based hybrid material has already been described previously [21]. However, we wanted to further demonstrate our ability to fabricate complex 3D shapes and thus have fabricated a micro-structure reminiscent of Zerg (StarCraft, Blizzard Entertainment^©^, Irvine, CA, USA)—see Figure 1. High volume suspended features were reproducibly fabricated, like the claws of the Zerg hydralisk. Literature shows that by precisely controlling the 3D cell microenvironments, one can achieve complex cellular responses, like stem cell homing towards artificially designed niches [31]. Another important group of factors in designing artificial cell niches are associated to the material chemistry and properties [32]. Integration of several materials in a single structure will hopefully one day help to guide stem cell differentiation towards different lineages on the same sample.

Multiple materials can be structured with laser two-photon polymerisation and the Al-based hybrid organometallic polymer presented in this work expands the selection of possible cellular niche materials even further. To date, a number of reports have been made of multiple-material structure fabrication using laser two-photon polymerization—including some of our own, in which PDMS, hybrid organometallic polymer based on Zr, PEG-DA and commercially-available OrmoComp have been integrated together [12]. Lamont et al. have developed a technique to exchange the material that is being used to fabricate structures on-the-go, allowing up to five different materials to be incorporated within the same structure with increased fabrication speed and potentially, multiple functionalities for applications like drug delivery, advanced optics, meta-materials and microrobotics [33]. Additionally, two or even more disparate fabrication techniques can be employed within the same structure as demonstrated with fused filament fabrication 3D-printed structure modification using laser ablation [34].

The addition of yet another 3D-printable material with new properties to the engineer’s menu will allow for a wider variety of new structures and applications to be envisaged, particularly in fields that require high precision micro-fabrication, like tissue engineering scaffolds and drug delivery devices.

### 3.2. Protein Adsorption Assay

Extracellular matrix protein adhesion to the different surfaces may affect the subsequent adhesion of cells to the surfaces. Collagen type I was chosen as a model system for this experiment—its adsorption to simple spin-coated samples was assessed due to collagen type I abundance in the extracellular matrix. Collagen supports cellular adhesion via RGD sequences, which are specifically recognised by cellular adhesion proteins integrins. These interactions facilitate strong integration between a tissue engineered scaffold and the surrounding tissues, so it is important to have a material that supports collagen adsorption to its surface well for applications where cell adhesion is beneficial.

Collagen itself has been used as a tissue engineering building block in various studies, for example that of Ber et al., where osteoblast growth was guided on 2D surfaces of collagen [35] or work by Ramanathan et al., who have investigated 3D hybrid collagen matrixes as antibacterial dermal substitutes [36]. Collagen and its derivatives are a widely investigated group of materials for soft tissue engineering, while for hard tissue, like bone, collagen coating is a highly desirable approach that can be used in conjunction with other materials. Multiple reviews have focused on the use of collagen as a tissue engineering material either in a pure or composite form [37,38,39]. Consequently, a possibility of coating hybrid polymer structures with collagen seems like a good approach for improving cellular integration and therefore chosen in this study.

In our experiments, the choice of protein quantification technique was limited by residual photo-initiators within the materials which rendered them fluorescent. Thus, the signal of fluorescence-based techniques for protein visualisation would have been hindered by sample autofluorescence. To overcome this issue, a colorimetric assay was employed.

**Figure 2:**
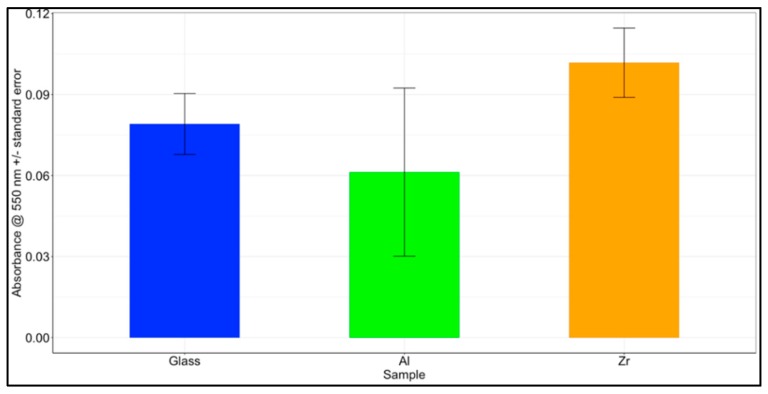
Collagen I adsorption to Al and Zr based hybrid organometallic polymers and glass. The results are presented as average Sirius Red absorbances + standard errors for three independent experiments with n = 3 per each experiment (a total of n = 9 per material).

Collagen adsorption was measured on both the hybrid polymers and reference glass slides by immersion in a solution of collagen, then dying the bound collagen and measuring the optical absorption of the dye. We found that even though the largest amount of collagen was found on the Zr surface and the smallest amount on Al surface, the results in each group were highly disperse, and no statistically significant differences were determined with *p* = 0.35 between Al and Zr, *p* = 0.72 between Glass and Zr and *p* = 0.81 between Glass and Al. The results are presented in Figure 2.

A calibration curve between the total amount of collagen I and Sirius Red absorption was prepared and is presented in Supplementary Materials Figure S1. A highly linear relation was found in the range of 0–100 μg with an R^2^ value of 0.994. The amounts of collagen adsorbed to the samples were calculated according to the linear model and were found to be 5.59 μg for Glass, 4.40 μg for Al and 6.73 μg for Zr on average per sample.

In our previous work [21], we investigated the contact angles of these materials and found them to be 39° for Glass, 72° for Al-based hybrid materials and 71° for Zr-based hybrid materials. The collagen adsorption data does not seem to correlate with the surface contact angles. Even though there is a significant difference between the contact angle of glass to Al or Zr surfaces, the difference in collagen I adsorption could not be distinguished due to a high deviation from the mean. A paper by Ying et al. [40] indicates that around a two-fold increase in the adsorption of collagen I to the glass surface is expected with an increase of the surface contact angle from 40° to 70°. An analogous result would be expected on other surfaces, since protein adsorption is governed by the same forces as surface contact angle—electrostatic and hydrophobic interactions.

### 3.3. Cell Viability

Having established the structurability of the materials as well as their collagen adsorption capacity, we investigated how their surfaces influence cellular viability. In this particular study, a primary rat muscle cell line was used to model a situation that would be as close as possible to a clinical one. Using primary muscle-derived stem cells is a safe choice in designing a possible future tissue engineering strategy due to the abundance of donor tissue sites, relatively mild donor site morbidity and high proliferative capacity as well as multipotency of the cells. A great example by Nieponice et al. demonstrates how rat muscle-derived stem cells could be used together with elastomeric scaffold-building materials in constructing vascular grafts [41]. The study successfully showed that the cells tended to home to the lumen of the vascular grafts and started to express α-actin, calponin as well as secrete collagen. Endothelial differentiation was supported via the presence of von Willebrand factor. Our isolated rat muscle-derived progenitor cells were positive for CD34, Myf5 and c-kit and negative for CD45 (Figure S2).

**Figure 3:**
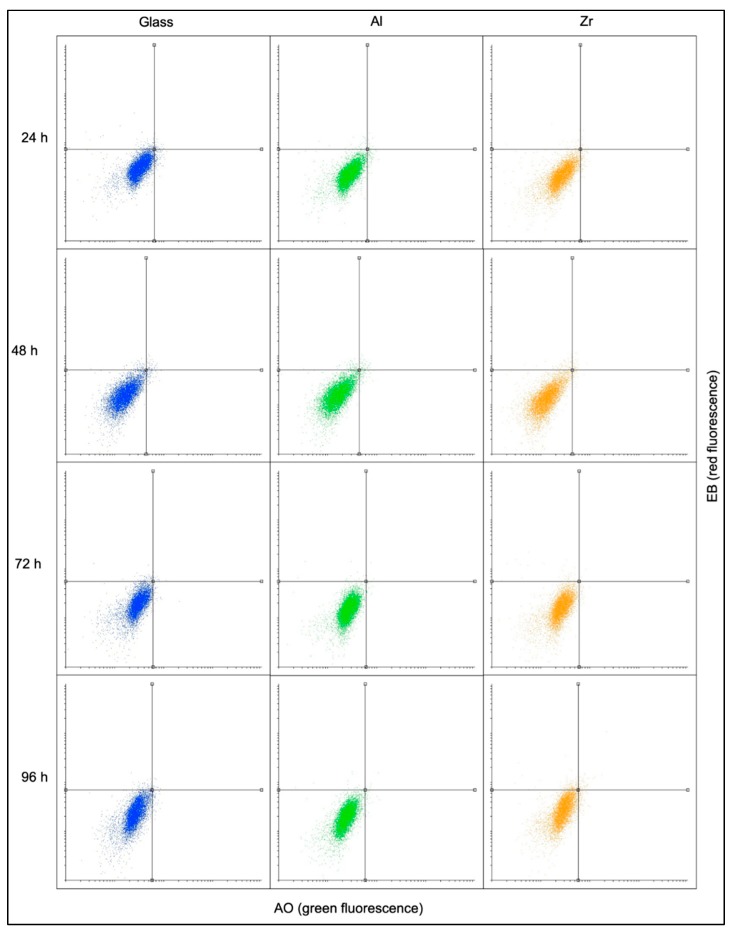
Flow cytometry data on cellular viability on Glass, Al and Zr surfaces after 24, 48, 72 and 96 h. The four quadrants in each case show the following: lower left—viable cells, upper left—necrotic cells, upper right—late apoptotic cells, lower right—early apoptotic cells. The lower left quadrant corresponds to 95% of all signals in all cases.

This experiment aimed to find the ratios between the numbers of viable, early apoptotic, necrotic and late apoptotic cells at different timepoints and on different surfaces. Flow cytometry data shows that no statistically significant differences between the numbers of cells in each category can be observed, since the majority of cells at each time point was viable (Figure 3). Additional data is presented in the Supplementary Materials, providing light microscopy images of cell culture at different time points as well as statistical analysis (Figure S3 and Figure S4 in the Supplementary Materials).

The results are in accord with our previous work on these materials with NIH/3T3 cells, which showed that the cells remain viable in culture for extended periods of time [21]. Data obtained in this experiment open the way for the future applications of both Al and Zr based hybrid materials. Long-term viability support of the cells is the first step towards engineering adequate implants for patients.

### 3.4. Cell Proliferation and Metabolism

As a subsequent step after viability assessment, the rate of cellular metabolism was measured using MTT assay. This test is a direct indicator of mitochondrial activity, since the dye is being digested by mitochondrial reductase to yield a colorimetric signal. This can be used as an indicator for overall cellular metabolism. It is important to know if cellular metabolism rates are different among the various surfaces, since significant deviations in cellular metabolism rates could potentially yield new properties of the cell population on that particular surface. Increased rates of metabolism could be associated with malignant formations and dysregulation of the cell cycle, while down-regulated rates of metabolism could indicate low cellular viability or transfer into a more quiescent state via differentiation or other mechanisms [42].

Light microscopy images show cells grown on both the hybrid polymer and glass surfaces to have formed confluent monolayers after 48 to 72 h (Supplementary Materials, Figure S3). No significant differences were observed neither in daily culture check-ups nor in the images taken of those cells. The cells had healthy spindle-shaped morphologies and stopped dividing after having reached confluency.

**Figure 4:**
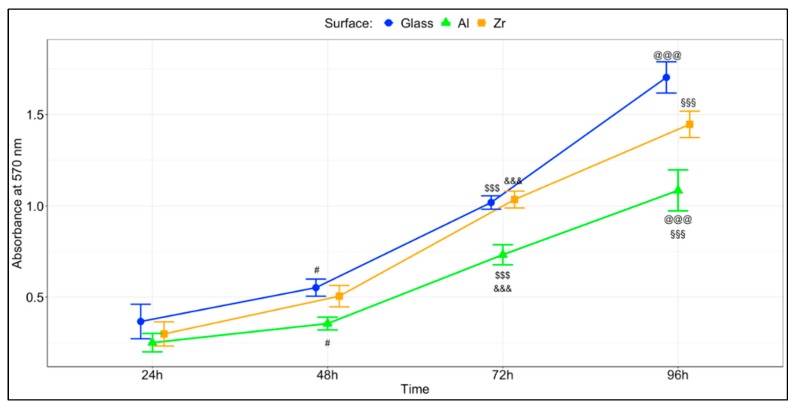
Cell proliferation as measured by MTT. The results are presented as average +/- standard error. A total of 9 measurements were performed per material per time point (3 independent experiments with 3 repetitions in each). #—significant difference between glass and Al with *p* < 0.05; $$$—significant difference between glass and Al with *p* < 0.001; &&&—significant difference between Al and Zr with *p* < 0.001; @@@—significant difference between glass and Al with *p* < 0.001; §§§—significant difference between Al and Zr with *p* < 0.001.

The assessment of total metabolic activity revealed that the metabolic rate of cells grown on glass was significantly higher than on the hybrid organometallic polymers after 96 h of culture (*p* < 0.001) and significantly higher than on the Al-based hybrid starting from 48 h (*p* < 0.05). These results are presented in Figure 4. Our previous study [21] investigated the proliferation rate of NIH/3T3 fibroblasts on analogous surfaces after 120 h and showed the Zr surface to support the highest rate, while no statistically significant differences were observed between Glass and Al.

The results obtained in this experiment show promise in using both the Al and Zr hybrid polymers in tissue engineering applications. Both materials supported cell growth, even though the total rate of metabolism was lower than on reference glass surfaces.

### 3.5. Cell Adhesion Strength

**Figure 5:**
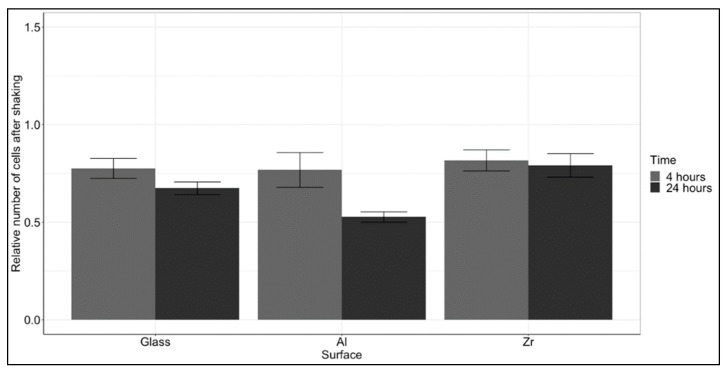
Evaluation of cell adhesion strength on the tested surfaces. The results are presented as a ratio between the average number of cells after shaking to that of cells before shaking after 4 and 24 h of culture + standard errors. A total of 9 measurements per material were performed (3 independent experiments with 3 repetitions each). No statistically significant differences were observed between the different time points on each surface.

Cellular viability, proliferation and adhesion are highly intertwined processes. Knowing that the viability does not change with surface and that the rate of cellular metabolism grown on the Al hybrid is lower, we sought to understand if the reduced metabolic activity was associated with weaker adhesion. The cells were seeded on the samples and then shaken vigorously at two timepoints. The number of remaining cells was counted and calculated against the number of cells on reference surfaces without shaking. The results are presented in Figure 5. There was a clear trend that showed a decrease in the number of adhered cells after 24 h, suggesting that the initial stage of attachment after 4 h was stronger. Even though there were substantial differences between the number of adhered cells after 24 h, the differences were not statistically significant. However, in spite of a lack of statistical significance, the number of cells that remained attached to the Al surface was the lowest and thus in accord with the proliferation data, that showed the lowest rate of MTT metabolism on the Al- based hybrid. Overall, the Al surface seems to be slightly less supportive of cell adhesion, though insignificantly. The reason for that could potentially be the lowest adsorption of collagen to the Al surface, as presented in Figure 2. To further investigate this difference, an experiment was devised to assess the expression of adhesion-associated proteins.

### 3.6. FAK/Akt Expression and Activation

When integrin-mediated cell adhesion takes place, a cascade of biochemical reactions is activated that delivers a signal about cellular attachment to the cell nucleus, which in turn activates the transcription and translation of certain proteins that are required for these processes. The cascade is activated when cell surface integrins dimerise after binding to a specific adhesion sequence in the ECM [43]. To initiate intracellular signalling integrin dimer induces conformational changes in focal adhesion kinase (FAK), which is one of the first proteins to join the newly forming focal adhesion. Subsequently, this leads to the recruitment of multiple other adhesion-associated proteins to the focal adhesion site. Upon formation of a focal adhesions, multiple pathways can be activated, one of which is the Akt pathway. This pathway is responsible for transferring a survival and proliferation signal to the nucleus via the mTOR pathway [44]. Akt can be activated by either phosphorylation on Ser473 or Thr308—this depends on the phosphorylating protein. In the case of integrin-mediated adhesion, PI3K is being activated and thus, only Ser473 is phosphorylated [45]. Phosphorylation on the Thr308 occurs from mTORC2, which is associated with cellular metabolism and cytoskeletal reorganisation.

**Figure 6:**
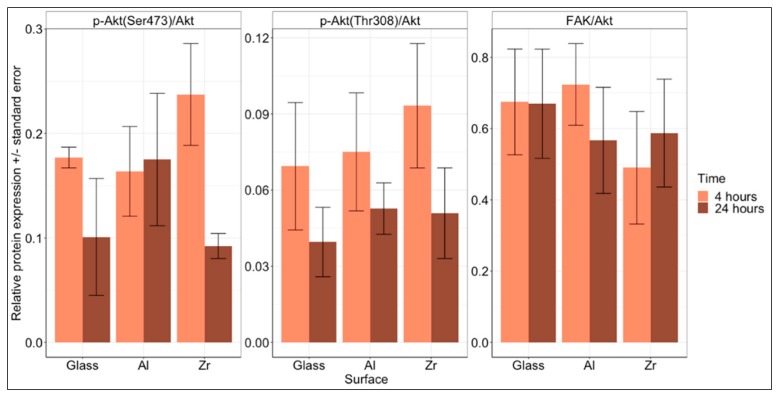
Western blot data, showing the expression of FAK and the expression and activation of Akt kinase after 4 and 24 h of culture on glass, Al and Zr surfaces. The amount of p-Akt (Ser 473), p-Akt (Thr308), Akt and FAK were observed. The results are presented as relative expression +/- standard error. Three independent experiments were performed. No statistically significant differences were observed.

To assess the expression and activation of adhesion-associated FAK pathway proteins, a Western blot analysis was performed on cells grown on the investigated Glass, Al and Zr surfaces. The results are presented in Figure 6 with membrane images presented in Supplementary Materials Figure S5. Even though no statistically significant results were observed on the investigated surfaces, a tendency for reduced phosphorylation of Akt kinase can be seen in most of the cases when comparing 4 and 24 h. This is to be expected as the adhesion-associated Akt kinase’s role is mostly pronounced during the initial cell-to-surface and cell-cell interactions that take place upon cell seeding.

The ratio between FAK and Akt kinases was mostly unchanged between time points and only slightly and insignificantly lower on Zr surfaces. This is again to be expected as the total amount of FAK and Akt kinases should remain unchanged.

On the glass surface, the cells tended to attach stronger during the initial 4 h—this is in accord with a higher level of Akt kinase activation in this time point. After 24 h the drop in the number of adhered cells and p-Akt (both Ser473 and Thr308) is clearly visible, though insignificant.

On the Al surface, the situation is similar in terms of p-Akt (Thr308), where the amount of phosphorylated Akt drops after 24 h in accord with a decreased number of cells. However, this was not observed in the p-Akt (Ser473) test. The level of FAK decreased as well, suggesting that the number of focal adhesions had been reduced as well.

The highest drop in terms of level of phosphorylation was observed on the Zr surface. From 4 to 24 h, the level of p-Akt (Ser473) dropped with a *p* = 0.25 and the level of p-Akt (Thr308) dropped with a *p* = 0.67. A slight, but insignificant increase was observed in the level of total FAK. Even though the changes in phosphorylation of Akt on Zr surfaces was the highest, the change in observed cell detachment was lowest in this case.

Even though some tendencies in cell attachment and adhesion-associated protein expression can be seen, these are not statistically significant.

Strong cellular adhesion is an essential prerequisite for successful tissue engineering techniques as discussed in a review by Lee et al. [46]. Whenever adhesion-associated signalling is insufficient, anchorage-dependent cells undergo anoikis, a form of programmed cell death that occurs in anchorage-dependent cells when they detach from the surrounding ECM.

### 3.7. Collagen Synthesis

Finally, we wanted to investigate whether the surface had any effect on the synthesis of collagen type I. Collagen is one of the main components of the extracellular matrix. Healthy tissues constantly undergo remodelling of their protein structures with the creation and digestion of new proteins by parenchymal cells, like fibroblasts. In tissue engineering, it is essential to have cells be able to model their environments by secreting ECM proteins, like collagen I.

**Figure 7:**
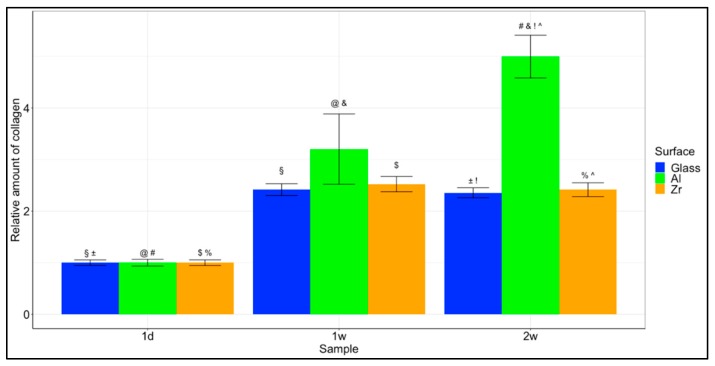
Collagen synthesis. Relative amount of collagen produced by cells grown on the different surfaces after 1 day, 1 week and 2 weeks. The results are presented as averages +/- standard errors. Statistically significant differences are indicated using different symbols. §—*p* < 0.05 between 1 day and 1 week glass; ±—*p* < 0.05 between 1 day and 2 week glass; @@@—*p* <0.001 between 1 day and 1 week Al; ###—*p* < 0.001 between 1 day and 2 week Al; $$—*p* < 0.01 between 1 day and 1 week Zr; %—*p* < 0.05 between 1 day and 2 week Zr; &&&—*p* < 0.001 between 1 week and 2 week Al; !!!—*p* < 0.001 between 2 week glass and 2 week Al; ^^^—*p* < 0.001 between 2 week Al and 2 week Zr. Other statistically significant differences (those between both different materials and different timepoints) are not presented.

Even though the adsorption was weakest to the Al-based hybrid and so was the cellular adhesion and proliferation, the synthesis of collagen in cells grown on the Al hybrid was the highest. The results are presented in Figure 7. The difference is already visible after one week, but it becomes significant after 2 weeks. This is to be expected as the process of extracellular matrix remodelling takes a substantial amount of time.

Other statistically significant differences were observed between Al surfaces after 1 day, 1 week and 2 weeks with *p* < 0.001 between all periods. As well as Zr surfaces after 1 day and 1 week with a *p* < 0.05.

The results show promise for tissue engineering applications, because they show a significant increase in the collagen produced in the cells grown on these surfaces. This means that the cells had not only attached to the surfaces, but they are comfortable and viable enough to start remodelling their environment via collagen synthesis.

## 4. Conclusions

We believe that the materials investigated in this work may be useful in constructing tissue engineered grafts of the future. This study provides a direct comparison between two materials that can be structured for laser two-photon polymerisation, assessing their support of primary rat myogenic cell growth. The differences between Zr and Al surfaces could potentially be attributed to a difference in adsorption of collagen, which in turn affects cellular adhesion strength, proliferation and the rate of extracellular matrix remodelling, but the results to support this are insignificant, suggesting a more thorough investigation into this process should be carried out.

An additional in vitro study should be performed in the future in order to evaluate how the adhesion-associated protein expression and activation changes over longer periods of time (several days or a couple of weeks) and whether that impacts cellular adhesion, viability and metabolism in a significant way.

Future studies in tissue engineering are likely to focus on integrating several materials with different properties in order to precisely guide cellular behaviour as well as investigating the role that artificial 3D niches with different geometries might play in affecting cellular differentiation and tissue maturation. As such, an extra option in the form of Al-containing hybrid organometallic polymer, presented in this work, is a choice, judging by its biocompatibility demonstrated in this study. In conjunction with its other advantages, like great 3D structuring capabilities, we believe the materials described in this work have potential to add value for the field of biomedical engineering.

## Supplementary Materials

The following are available online at https://www.mdpi.com/1996-1944/12/23/3932/s1, Figure S1: A curve of collagen I content vs Sirius Red absorbance at 550 nm. An average of three independent experiments with n = 3 in each is presented ± standard deviation. Regression analysis was performed, showing that the dependence is linear in the measurement range (0–100 μg) with R^2^ = 0.994 and f(x) = 0.0199x−0.0322, Figure S2: Characterization of rat skeletal muscle-derived progenitor cells. Fluorescent microscope images of cells immunostained for CD45, CD34, c-kit and Myf5. The scale bars correspond to 100 microns, Figure S3: Representative images of cells grown on glass, Al and Zr surfaces after 24, 48, 72 and 96 h. The scale bars correspond to 100 microns, Figure S4: Flow cytometry data on cellular viability on Glass, Al and Zr surfaces after 24, 48, 72 and 96 h. The results are presented as average absorbance +/- standard deviation, derived from 3 independent experiments with n = 3 per experiment. No statistically significant differences were found between the surfaces, Figure S5: Images of the Western blot membranes, showing the expression levels of p-Akt (Ser473), p-Akt (Thr308), Akt and FAK proteins. Tubulin was used as a reference. From left to right: 1st membrane: ladder, Al(4h), Al(24 h), Zr(4 h), Zr(24 h), Glass(4 h), Glass(24 h), Al(4 h), Al(24 h), Zr(4 h); 2nd membrane: ladder, Zr(24 h), Glass(4 h), Glass(24 h), Al(4 h), Al(24h), Zr(4 h), Zr(24 h), Glass(4 h), Glass(24 h).
