# Supplementary material for: Biocompatibility Investigation of Hybrid Organometallic Polymers for Sub-Micron 3D Printing via Laser Two-Photon Polymerisation
Source: Materials (Basel). 2019 Nov 27;12(23):3932. doi: 10.3390/ma12233932 (PMC6926539; doi:10.3390/ma12233932)
Supplement: Supplementary file 1 [file materials-12-03932-s001.pdf]

materials

Article

Biocompatibility Investigation of Hybrid Organometallic Polymers for Sub-Micron 3D Printing via Laser Two-Photon Polymerisation

Evaldas Balčiūnas [1], Nadežda Dreižė [1], Monika Grubliauskaitė [1], Silvija Urnikytė [1], Egidijus Šimoliūnas [1], Virginija Bukelskienė [1], Mindaugas Valius [1], Sara J. Baldock [2,3], John G. Hardy [2,3,*] and Daiva Baltriukienė [1,*]

1 Institute of Biochemistry, Life Sciences Centre, Vilnius University, Vilnius 10257, Lithuania; ev.balciunas@gmail.com (E.B.); nadezda.dreize@gmail.com (N.D.); monika.grub@gmail.com (M.G.); urniksi@gmail.com (S.U.); egidijus.simoliunas@gmail.com (E.S.); virginija.bukelskiene@bchi.vu.lt (V.B.); mindaugas.valius@bchi.vu.lt (M.V.)

2 Department of Chemistry, Lancaster University, Lancaster LA1 4YB, UK; s.baldock@lancaster.ac.uk

3 Materials Science Institute, Lancaster University, Lancaster LA1 4YB, UK

* Correspondence: j.g.hardy@lancaster.ac.uk (J.G.H.); daiva.baltriukiene@bchi.vu.lt (D.B.); Tel.: +370-5223-4379 (D.B.)

Received: 29 October 2019; Accepted: 24 November 2019; Published: date

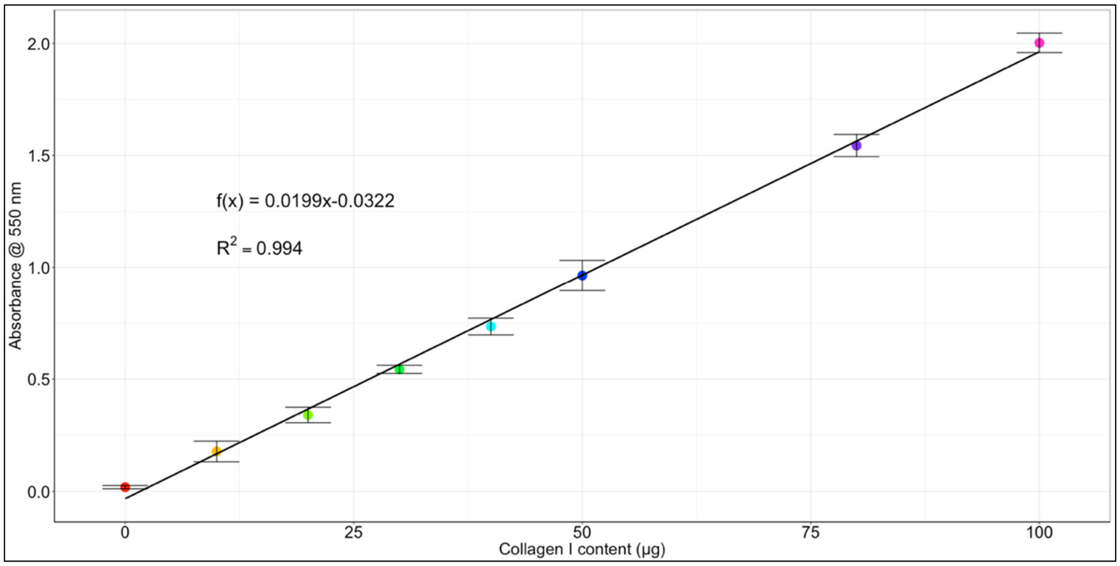

Figure S1. A curve of collagen I content vs Sirius Red absorbance at 550 nm. An average of three independent experiments with n = 3 in each is presented ± standard deviation. Regression analysis was performed, showing that the dependence is linear in the measurement range (0–100 µg) with $R^2 = 0.994$ and $f(x) = 0.0199x-0.0322$.

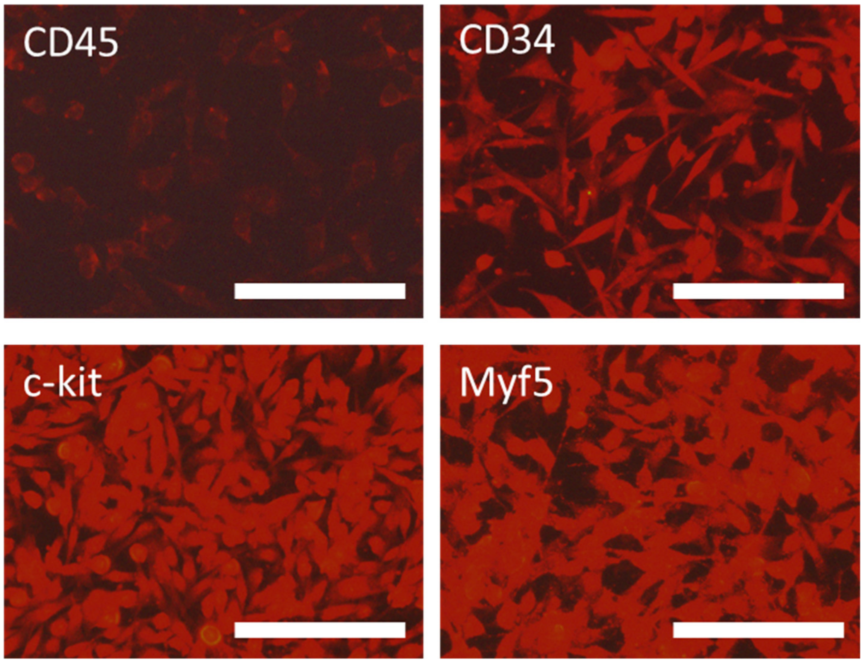

Figure S2. Characterization of rat skeletal muscle-derived progenitor cells. Fluorescent microscope images of cells immunostained for CD45, CD34, c-kit and Myf5. The scale bars correspond to 100 microns.

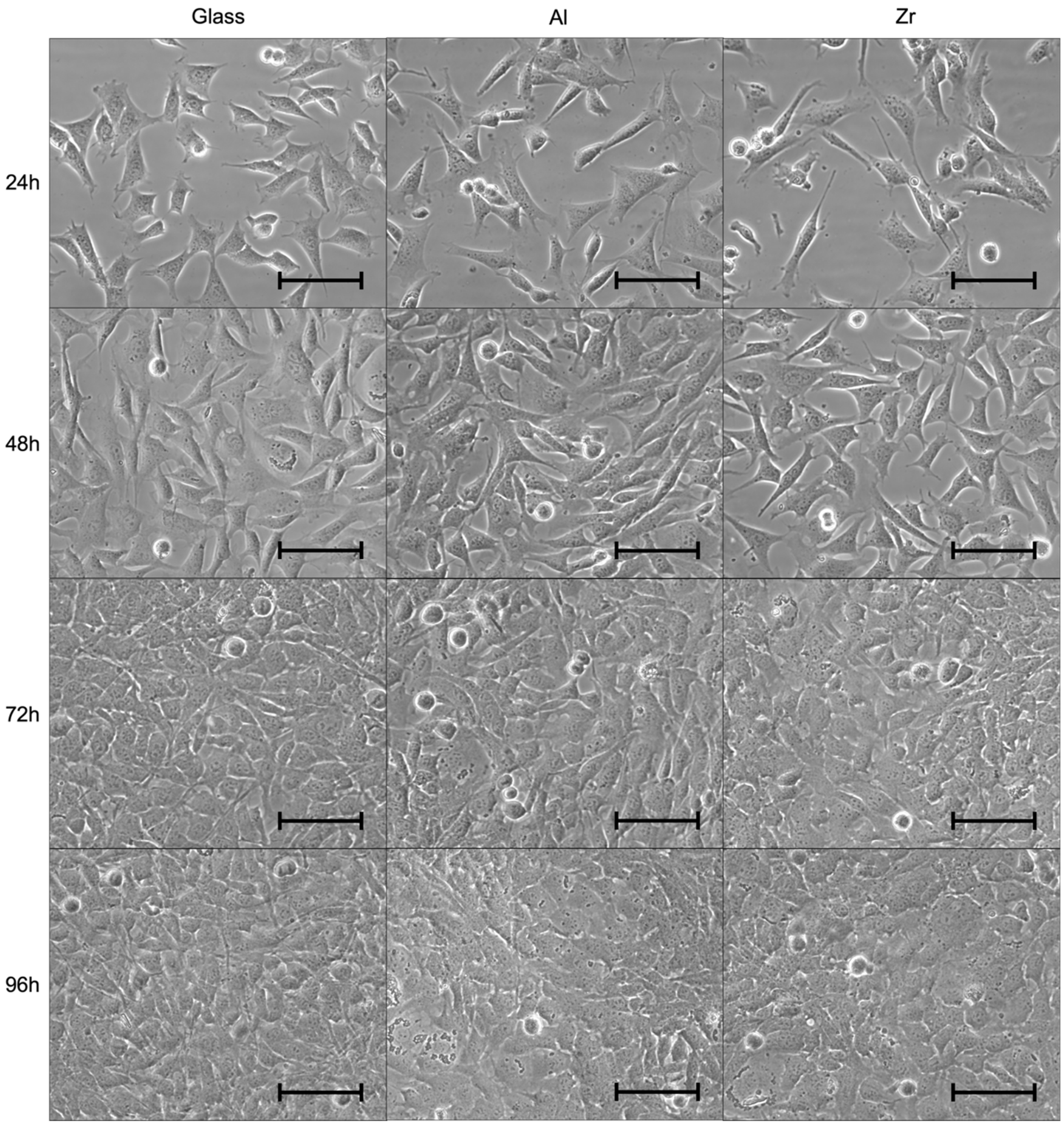

Figure S3. Representative images of cells grown on glass, Al and Zr surfaces after 24, 48, 72 and 96 h. The scale bars correspond to 100 microns.

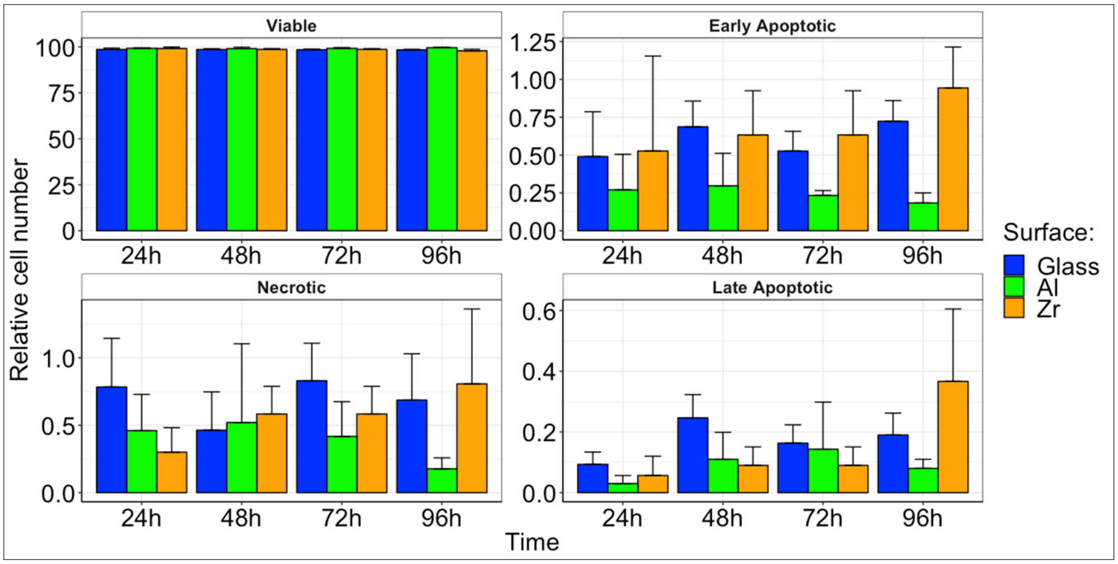

Figure S4. Flow cytometry data on cellular viability on Glass, Al and Zr surfaces after 24, 48, 72 and 96 h. The results are presented as average absorbance +/- standard deviation, derived from 3 independent experiments with n = 3 per experiment. No statistically significant differences were found between the surfaces.

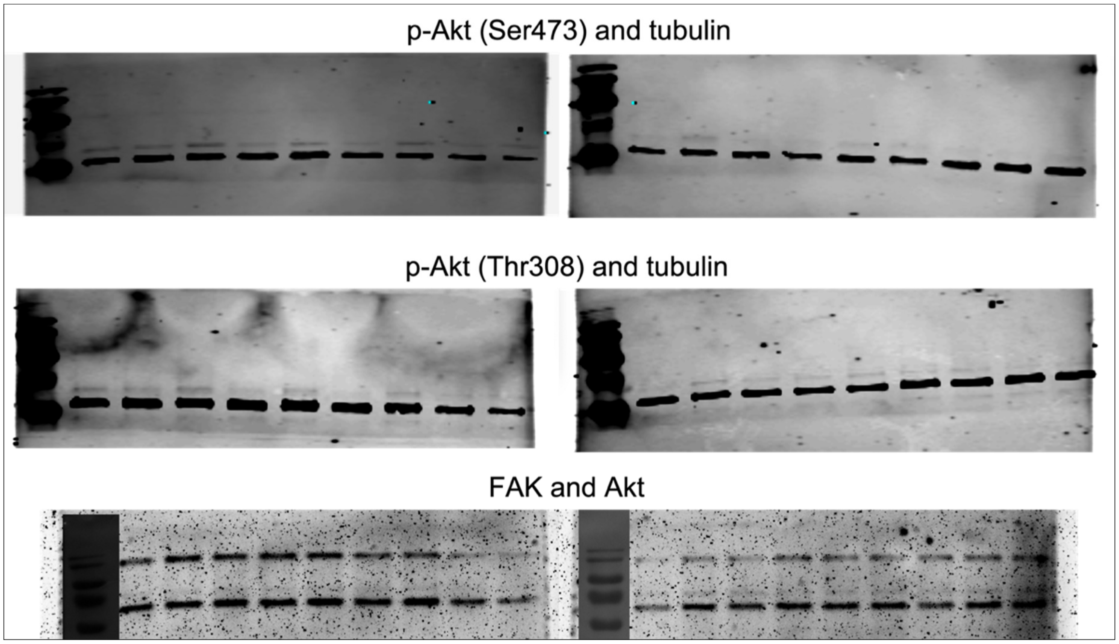

Figure S5. Images of the Western blot membranes, showing the expression levels of p-Akt (Ser473), p-Akt (Thr308), Akt and FAK proteins. Tubulin was used as a reference. From left to right: 1st membrane: ladder, Al(4h), Al(24 h), Zr(4 h), Zr(24 h), Glass(4 h), Glass(24 h), Al(4 h), Al(24 h), Zr(4 h); 2nd membrane: ladder, Zr(24 h), Glass(4 h), Glass(24 h), Al(4 h), Al(24h), Zr(4 h), Zr(24 h), Glass(4 h), Glass(24 h).
